# Successful Oocyte Retrieval After Follicular Fluid Aspiration in Suspicious of Ovarian Torsion

**DOI:** 10.7759/cureus.12192

**Published:** 2020-12-20

**Authors:** Daichi Inoue, Yoshimasa Asada

**Affiliations:** 1 Assited Reproductive Technology, Asada Ladies Clinic, Nagoya, JPN; 2 Assisted Reproductive Technology, Asada Ladies Clinic, Nagoya, JPN

**Keywords:** controlled ovarian stimulation, ovarian torsion, pregnancy, follicle aspiration, volume reduction

## Abstract

During controlled ovarian stimulation, a 34-year-old woman complained of right lower abdominal pain after the decision to retrieve oocytes. Ovarian torsion was suspected and confirmed, so aspiration of follicular fluid was performed prior to oocyte retrieval for volume reduction of the affected ovary. Two days after that, oocytes were successfully collected. Four months later, the frozen embryo was transferred and got pregnant. In conclusion, it is possible to perform volume reduction before ovum pick up (OPU), and also possible to become pregnant by embryo transfer afterward. This is the rare case report of follicular aspiration prior to oocyte retrieval.

## Introduction

Surgical treatment is the first option in the treatment of ovarian torsion [1]. However, if the onset of torsion is suspected before oocyte retrieval, it is challenging, especially if the facility for major surgery is restricted. In this report, ovarian torsion was suspected two days before oocyte retrieval, and follicular fluid was aspirated for volume reduction. The decompression was successful, and the symptoms were alleviated, so the oocytes were successfully collected two days later. Four months later, a frozen-thawed embryo transfer was performed, and the patient became pregnant.

## Case presentation

The first consultation with assisted reproductive technology

A 34-year-old woman with no previous pregnancy or significant past medical history visited our hospital with a chief complaint of infertility for six months. Menstrual cycles were irregular with a duration ranging from 25 - 32 days. Her body mass index was 23.1 kg/m^2^. The serum concentration of prolactin was 15.6 ng/mL, and the serum concentration of anti-Müllerian hormone was 2.89 ng/mL. After the failure of one cycle of intrauterine insemination, due to the patient's wishes, the decision was made to step up to assisted reproductive technology treatment.

Clinical course during controlled ovarian stimulation

From cycle day (CD) four, follicular stimulating hormone (Gonalef®; Merck, Tokyo, Japan) 300 IU x 4 days and human menopausal gonadotropin (hMG injection - Teizo®; Aska Pharmaceutical, Tokyo, Japan) 300 IU x 10 days were administered, and from controlled ovarian stimulation (COS) on day 8, in flexible method, a total of four doses of Cetrotide® (cetrorelix acetate; Merck Biopharma, Tokyo, Japan) 0.25 mg subcutaneous administration (A in Table 1 means the dosage of 0.25 mg). On COS day 13, oocyte retrieval was decided three days later (Table 1).

**Table 1:** Clinical course during controlled ovarian stimulation From top to bottom, menses cycle day, stimulation day from the start, the dosage of drugs (FSH - follicular stimulating hormone (IU); hMG - human menopausal gonadotropin (IU); hCG - human chorionic gonadotropin (IU); A - cetrotide 0.25mg). The diameter of up to four large follicles (mm) and number of follicles (n) (Rt - right ovary; Lt - left ovary, 1 - 4; 4th largest follicle diameter; f - number of follicle ≥ 5 mm, ≥ 14mm in parenthesis) EM - thickness of endometrium (mm) Serum concentration (FSH - follicular stimulating hormone (mIU/mL); LH - luteinizing hormone (mIU/mL); E2 - serum estradiol (pg/mL) [if the blood concentration has been decreased from the previous time, it is marked with asterisk]; hCG - human chorionic gonadotropin (mIU/mL); P4 - serum progesterone (ng/mL) [if the blood concentration is over 2 ng/mL, it is marked with asterisk]).

Cycle day		4	5	6	7	8	9	10	11	12	13	14	15	16	17	18	19
Stimulation day		1	2	3	4	5	6	7	8	9	10	11	12	13	14	15	16
FSH		300	300	300	300												
hMG						300	300	300	300	300	300	300	300	300	300		
Low dose hCG										30	30	30	30	30	30		
Antagonist									A		A		A	A			
Rt	1					15x11			26x19					58x24			
	2					13x11			16x19					36x33			
	3					12x9			17x15					31x26			
	4					10x7			17x14					30x24			
	f	5				9			14(5)					18(9)			
Lt	1					10x11			15x16					36x23			
	2					9x7			15x14					30x25			
	3					9x7			14x12					29x22			
	4					8x7			14x11					25x23			
	f	4				7			12(2)					16(12)			
EM		4.5				10.3			10.1					6.6			
FSH		5.4				12.2			17.9					17.2			11.6
LH		4.4				2.1			2.5					<0.1			5.5
E2		24				1227			5255					15574	12194*		1765*
hCG		<0.5												3.6	2.6		
P4															2.44*		6.81*

Trial of volume reduction

**Figure 1:**
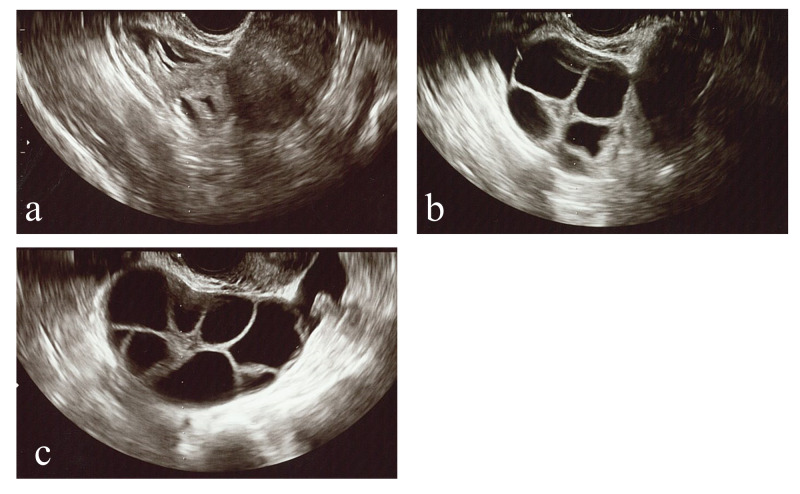
Echogram of bilateral ovary before and after puncture a: right ovary after the aspiration of follicular fluid b: right ovary before the oocyte retrieval c: left ovary before the oocyte retrieval

The day after the final consultation, she visited the hospital with intermittent right lower abdominal pain and mild nausea. Estradiol was lower than the previous day, and progesterone was raised. The possibility of ovulation pain was also suggested, but the luteinizing hormone on the previous day was <0.1 mIU/mL, and no findings of ovulation or luteinization by ultrasound echogram were found. Physical examination showed that tenderness and rebound pain around the right ovary and blood pressure was 118/76 mmHg, pulse was 80 beats per minute. Since there was no evidence of inflammation, we suspected pedicle torsion of the right ovary. Because computed tomography, magnetic resonance imaging, and other image diagnostics could not be performed due to facility limitations, volume reduction by follicular fluid aspiration was suggested to be performed with consent, and if the symptoms are alleviated, a deep observation would be performed after oocyte retrieval. Under intravenous anesthesia, follicular fluid of the right ovary, which is the affected side, was aspirated (Figure 1). As a precaution, the eggs were searched, but no cumulus-oocyte complex was found in the follicular fluid. Follicular fluid was found yellow and transparent. After the awakening from anesthesia, mild lower abdominal pain was still observed, but as a final oocyte maturation trigger, gonadotropin-releasing hormone (GnRH) agonist (leuprolide acetate - Lucrin®; AbbVie, Illinois, USA) was administered (subcutaneous administration, 1 mg) and 35 hours later, the oocytes were retrieved with a transvaginal needle under intravenous anesthesia.

Result of oocyte retrieval and fertilization

On the day of ovum pick up (OPU), estradiol decreased to 1765 pg/mL, and progesterone increased to 6.81 ng/mL. Fifteen oocytes (six from the right ovary, nine from the left ovary) were collected, and all of them were meiosis II stage, so fertilization was split. Intracytoplasmic sperm injection (ICSI) for 11 and conventional in vitro fertilization (IVF) for four were performed, respectively. Fertilization results were two pronuclear (2PN) x 10 from ICSI and 2PN x 4 from conventional IVF. According to our hospital's policy, 2PN x 8 were cryopreserved, and six were cultured in blastocysts. As a result, two blastocysts (4BA, 4CB) were obtained.

Embryo transfer and pregnancy

At the patient's request, after four months of OPU, two blastocysts were transplanted at a hormone replacement cycle, and luteal support was performed with chlormadinone acetate (Lutoral™; Fujipharma, Tokyo, Japan). The pregnancy was diagnosed 12 days after the transplantation (gestational age; four weeks +3). Serum hCG was 805 IU/L.

The gestational sac was detected by ultrasound at five weeks +3. We followed up to nine weeks at this hospital, confirmed Crown-rump length 23.4 mm, and identified fetal heartbeat, and she was referred to another hospital for pregnancy management.

## Discussion

Ovarian torsion is a complete or partial rotation of the vascular pedicle of the ovary, impeding the blood flow of the ovary. Diagnosis of ovarian torsion is often difficult and must be based on history, clinical findings, and ultrasonography [2]. The pain is usually severe and persistent, but if the torsion is partial and intermittent, the pain is diminishing. There are also autonomic reflexes, such as nausea, vomiting, and tachycardia [1]. Also, the existence of blood flow by Doppler does not necessarily deny the torsion. In addition, it has been reported that CT is not essential in the meta-analysis, and the diagnostic power of CT is said to be the same as that of ultrasound [3, 4].

The patient, in this case, complained of lower abdominal pain two days before oocyte retrieval, but physical findings showed tenderness and rebound pain in the right adnexal region, which probably occurred due to ovarian swelling, tension, and stretching of ligaments following ovarian stimulation at first. The differential diagnosis was follicle rupture (or ovulation) and hemorrhage [5], but transvaginal ultrasound findings ruled out the other diagnosis, and pedicle torsion was suspected by the elimination.

If ovarian torsion is suspected, a surgical method such as laparoscopic surgery or laparotomy is considered to be a diagnostic treatment. When ovarian torsion is suspected, the persistence of symptoms over 48 hours determines whether the ovaries can survive or not [1, 6].

Contrary to this, there are reports of using sildenafil citrate to reduce ovarian edema and improve venous blood flow without surgical treatment for ovarian torsion after OPU. The use of sildenafil citrate resulted in a sustained return of ovarian tissue, which helped the ovary's natural detorsion without surgical intervention [7].

Regarding the relationship between ovarian torsion and infertility treatment, Romanski et al. reported that infertility treatment using gonadotropin treatment to control ovarian stimulation is an independent risk factor for ovarian torsion [8]. It has also been suggested that there is a relationship between peak serum estradiol (E2) levels, ovarian diameter, and ovarian volvulus.

As a predictor of ovarian torsion, there is a report that the maximum ovary diameter is 50 mm. The sensitivity when the threshold was 50 mm was 91% (83-97%) [9], and in this case, it was also suspected to be ovarian torsion based on the size of the right ovary.

Although it was difficult to determine whether the drop of serum estradiol was the effect of oocyte maturation trigger or the effect of follicular fluid aspiration, oocyte retrieval was performed under intravenous anesthesia.

There are various reports on the role of follicular fluid - follicular growth, ovulation, oocyte nutrition, maturation and oocyte quality, sperm capacitation, fertilization, early embryonic development, and so on [10, 11]. It was discussed with the patient about adverse effects caused by puncture and aspiration of follicular fluid before OPU, such as inability to retrieve oocyte, decrease maturation rate or fertilization rate, and effects on embryonic development. The symptom is alleviated by fine-needle aspiration, and the follicles can be safely recovered after two days because the follicles have almost returned to their original size.

## Conclusions

Ovarian torsion associated with infertility treatment often occurs mainly with ovarian hyperstimulation syndrome (OHSS) after OPU, but if it seems to be caused by ovarian swelling just before OPU. It is possible to perform volume reduction before OPU and also possible to become pregnant by embryo transfer afterward.
